# Losartan Attenuates Radiation-Induced Damage on Testes and Accelerates Tubular Regeneration

**DOI:** 10.3389/frph.2022.904804

**Published:** 2022-07-14

**Authors:** Lais L. Manção dos Santos, Marco G. Alves, Agnaldo Bruno Chies, Maria Angélica Spadella

**Affiliations:** ^1^Laboratory of Human Embryology, Marília Medical School – FAMEMA, Marília, Brazil; ^2^Unit for Multidisciplinary Research in Biomedicine (UMIB) and Institute of Biomedical Sciences Abel Salazar, University of Porto, Porto, Portugal; ^3^Biotechnology of Animal and Human Reproduction (TechnoSperm), Institute of Food and Agricultural Technology and Unit of Cell Biology, Department of Biology, Faculty of Sciences, University of Girona, Girona, Spain; ^4^Laboratory of Pharmacology, Marília Medical School – FAMEMA, Marília, Brazil

**Keywords:** Ang II receptor (type 1) blocker, cancer, germ cells, radioprotection, radiotherapy, spermatogenesis

## Abstract

Male germ cells are particularly susceptible to radiation; infertility being a common consequence after radiotherapy as it impairs spermatogenesis. This study aimed to test whether treatment with losartan (LOS), a selective antagonist of angiotensin II receptor subtype 1 (AT1R), can prevent or attenuate the acute and long-term radiation-induced damage to testes. Wistar rats were randomly distributed into six groups, three of which were studied on day 2 after irradiation: control (CTRL 2), irradiated non-treated (IR 2), and irradiated and treated with LOS (IRLOS 2); and three other groups that were studied on day 60 after irradiation: control (CTRL 60), irradiated non-treated (IR 60), and irradiated and treated with LOS (IRLOS 60). Seven consecutive days before and on the day of irradiation with 2.5 Gy directly administered in the scrotum, the animals were treated with LOS (34 mg/kg/two times/day). This treatment was continued 2 or 60 days after irradiation. The sperm quality was assessed from epididymis cauda. In addition, the testes were submitted to histopathological and morphometric-stereological analysis as well as the proliferating cell nuclear antigen (PCNA) quantification. Serum FSH and LH and plasma testosterone levels were also determined. The data obtained 2 days after the irradiation showed germ cell apoptosis, formation of vacuoles in the seminiferous epithelium, sloughing of germ cells into the lumen, and retention and phagocytosis of step-19 spermatids in Sertoli basal cytoplasm. The treatment with LOS in this period did not prevent or attenuate a radio-induced damage to the testes, illustrating that this drug does not protect against apoptosis derived from direct effects of radiation. On the other hand, 60 days after exposure, the data evidenced the deleterious effects of ionizing radiation on the testes as decreasing of testicular, epididymal, and seminal vesicle masses; tubular atrophy; reduction of cellular proliferation; and loss of germ cells. LOS was able to prevent some of those deleterious effects, promoting improvements in seminal vesicle mass, sperm vitality, plasma testosterone levels, vacuole number, and cell proliferation. In conclusion, inhibition of the AngII/AT1R axis by LOS is effective in protecting the indirect/delayed radiation damage resulting from oxidative stress established in the tissue.

## Introduction

Faced with the increasing worldwide incidence of cancer cases in the coming decade (1), radiotherapy is becoming an important therapeutic resource, especially to treat prostate cancer and other pelvic cancers. It is based on the use of ionizing radiation to destroy or prevent the tumor cells' multiplication, promoting the control of the disease or even its cure (2, 3). In addition to the effects on the tumor, however, radiotherapy also damage healthy adjacent tissues through direct and indirect molecular events related to oxidative stress (2, 4). Thus, radiotherapy may affect the male reproductive capacity, causing temporary, prolonged, or even definitive azoospermia, depending on the treatment time and radiation dose (5–7).

Male germ cells are particularly susceptible to radiation effects; thus, infertility is a common consequence of total or partial body irradiation, since it impairs the cyclical process of spermatogenesis (8, 9). This harmful effect on the spermatogenic epithelium eliminates differentiated spermatogonia as well as decreases the number of germ cells in advanced stages of the spermatogenesis, thereby resulting in reduced sperm production and other gamete's intrinsic problems (10–12). Thus, investigating irradiated testes is important by its common exposition, direct or indirectly, in radiotherapy procedures.

Acute biological effects in irradiated tissues may involve direct interaction of ionizing radiation with DNA that controls vital cellular functions. It is believed that this mechanism may represent up to 30% of the total radiation exposure effect (4). On the other hand, radiation may also act indirectly since its energy is first transferred to an intermediate molecule, such as water, causing radiolysis, thereby leading to the formation of free radicals and consequent cell injury or death. These radiolysis products, such as superoxide, hydrogen peroxide, and radical hydroxyl, may target vital cellular structures since they are highly reactive (13).

Renin-angiotensin system (RAS) may also be involved in the radiation-induced cell damage and, consequently, dysfunction of irradiated tissues. Previous studies show RAS participation in the indirect effects of free radicals generated by radiation (14–18). Notably, biological effects of both ionizing radiation and angiotensin II/angiotensin receptor subtype 1 (Ang II/AT1R) axis are mediated directly or even indirectly, through upregulation of profibrogenic and proinflammatory pathways and by the formation of reactive oxygen species (ROS) *via* activation of NADPH oxidase, triggering cellular damage (16, 17). Previous studies also show that pharmacological inhibition of RAS may prevent or improve cancer-treatment-induced adverse events, such as pneumonitis, nephropathies, cardiotoxicity, tumor cachexia, and vasogenic edema, in both humans and animals, probably through attenuation of oxidative stress (19–23). The effectiveness of RAS block to prevent/reduce radiation-induced harmful effects is due to inhibition of the activity of locally produced Ang II. It is believed that these RAS actions do not involve modulation of the classical vasoconstrictor function of Ang II, but the inhibition of its proinflammatory and profibrotic genic actions (10, 16, 22). Since an intrinsic RAS has already been described in the reproductive organs (24–27), our research group investigated the radioprotective role of losartan and telmisartan, selective antagonists of AT1R (28–30). The obtained results revealed that AT1R antagonism accelerates the recovery of the seminiferous epithelium, indicating a potential radioprotective effect. The employed radiation dose (5.0 Gy) was high, however, which made the complete regeneration of the seminiferous epithelium after treatment with these AT1R antagonists impossible.

Herein, we propose to further explore a new experimental protocol to test the radioprotector properties of AT1R antagonists, administrating a radiation dose that induces about 50% of harmful effects upon the testes (31). In this condition, this study aimed to assess whether treatment with losartan can prevent/attenuate the acute and delayed damage induced by ionizing radiation on the testes.

## Materials and Methods

### Animals

Forty-seven male *Wistar* rats (350–400 g), 12 weeks old, were obtained from the Central Vivarium of Marília Medical School (FAMEMA), Marília, São Paulo State, Brazil. The animals were maintained in polypropylene cages (50 cm x 40 cm x 20 cm), 3 animals per cage, under a 12-h light/12-h dark cycle at a controlled temperature (23 °C ± 1 °C), with free access to water and pelleted rodent chow. This study was approved by the Ethics Committee for the Use of Experimental Animals from the Marília Medical School (CEUA/FAMEMA, protocol number 1525/19) and conducted according to the National Council of Animal Experimentation Control (CONCEA, Brazil).

### Study Design and Experimental Groups

The experiments were performed in two distinct moments: (1) 2 days post-irradiation to approach the acute effects of the ionizing radiation on the testes and (2) 60 days post-irradiation to approach late damage, considering the spermatogenic cycle in rats, which is of approximately 58 days, being a constant cycle (32). Thereby, the rats were randomly distributed in six experimental groups of eight animals: *CTRL 2* (control), non-irradiated and non-treated, euthanized after 2 days post-irradiation; *IR 2*, irradiated and non-treated, euthanized 2 days after irradiation; *IRLOS 2*, irradiated and treated with losartan, euthanized 2 days after irradiation; *CTRL 60* (control), non-irradiated and non-treated, euthanized after 60 days post-irradiation; *IR 60*, irradiated and non-treated, euthanized 60 days after irradiation; *IRLOS 60*, irradiated and treated with losartan, euthanized 60 days after irradiation.

### Irradiation Protocol

The rats were weighed and then anesthetized with tribromoetanol (25 mg/100 g; i.p.; Sigma-Aldrich^®^, MO, USA) and immobilized upon wooden support. The scrotum was carefully tied with a bow of string and positioned in a casing surrounded by paraffin to assure the uniformity of irradiation dose delivery in the organ. The radiation (single dose of 2.5 Gy administered at a dose rate of 1.05 Gy/min) was delivered from a Clinic 6EX linear accelerator at 6MV (Varian, California, USA), at a distance of 100 cm, surrounding the 5 x 5 cm scrotal field in the anteroposterior direction and in the supine position at a depth of 2.0 cm. All the control animals were submitted to the same procedures except for the exposure to ionizing radiation. For this study, the chosen dose to be applied directly to the scrotum of the animals was previously established as described by Spadella et al. (31), based on a dose-response curve (radiation dose vs. radiation-induced effects on reproductive tissue).

### Treatment

The animals were treated with losartan (Gemini, Goiás, Brazil), 34 mg/kg twice a day, administered by gavage. Before each administration, 0.5% losartan solution was freshly diluted in carboxymethylcellulose (Denver Especialidades Químicas Ltda, São Paulo, Brazil). The treatment with losartan started 7 consecutive days before and on the day of the irradiation exposure and it was continued for 2 and 60 consecutive days to evaluate the effects of the treatment on the acute and late damage to the testes, respectively. This losartan dosage does not cause gonadotoxicity and was based on the previous study of our research group performed on rats (30). The control animals and those only exposed to ionizing radiation received only the vehicle twice daily.

### Blood and Tissue Harvest

Two or 60 days after irradiation, the rats were weighed and euthanized by carbon dioxide (CO_2_) inhalation followed by exsanguination. Blood samples were collected *via* inferior cava vena puncture with the BD Vacutainer^®^ blood collection tubes (Serum [Ref 367820], K2 EDTA [Ref 367861], Becton, Dickinson and Company, USA) for serum FSH/LH and plasma testosterone measurement. These harvested blood samples were then centrifuged for 20 min at 4°C (3.500 rpm) to obtain plasma and serum, which were stored in a freezer at −80°C until the hormonal determinations. In addition, the testes, the epididymis, the prostate, and the seminal vesicles were harvested and weighted. All wet weights (in g) were standardized by the respective body weight of the animal (in kg).

### FSH, LH, and Testosterone Level Determination

The serum FSH/LH and plasma testosterone levels were determined by enzyme immunoassay using FSH [RE52121, analytical sensitivity 0.86 mlU/ml], LH [RE52101, analytical sensitivity 1.27 mlU/ml], and testosterone [RE52151, analytical sensitivity 0.12 ng/ml] ELISA method, according to the manufacturer's instructions (IBL International, Germany).

### Histopathological Analysis of the Testis and Epididymis

For histological studies, the testes and the epididymis were fixed in 2% glutaraldehyde and 4% paraformaldehyde (MERK, Germany) in 0.1 M of Sorensen's phosphate buffer at pH 7.4 for 24 h. The materials were then washed for 24 h, dehydrated in alcohol, and embedded in a Leica Historesin Embedding Kit as previously described (31). The 3-μm-thick sections were stained with hematoxylin and eosin. The analysis of general morphology was conducted according to the recommendations of Foley (33), Lanning (34), and Kempinas and Klinefelter (35). Micrographs were obtained using an Olympus DP-25 digital camera attached to an Olympus BX41 microscope. The CellSens Olympus capture software was used (Olympus, Tokyo, Japan).

### Morphometry and Stereology of the Testis

The morphometric and stereological analyses were conducted as described by Spadella (31), from 10 histological fields per testis, according to adapted stereology protocol (36). Briefly, the tubular diameter (μm) of at least one hundred seminiferous tubules in stage IX of spermatogenesis per animal was measured using the closed polygon tool of the CellSens Olympus software by Dimension (Olympus, Toquio, Japan). For the stereology, a 169-intersection grid was projected onto each histological field to determine the density (D) present in the testes of the seminiferous tubules (ST), interstitial tissue (IT), and vacuoles (V) in the seminiferous epithelium. The densities (D) occupied by each testicular component were obtained, considering the formulas


$$\begin{array}{l} \text{D(ST)}=\frac{\text{total}\;\text{number}\;\text{of}\;\text{ST}\;\text{counted}\;}{169}\;.\;100\\ \text{D(IT)}=\frac{\text{total}\;\text{number}\;\text{of}\;\text{IT}\;\text{counted}\;}{169}\;.\;100\\ \text{D(V)}=\frac{\text{total}\;\text{number}\;\text{of}\;\text{V}\;\text{counted}\;}{169}\;.\;100 \end{array}$$

### Sperm Evaluation

The right epididymis was dissected and placed on a Petri dish containing 5 ml of pre-warmed (37°C) phosphate-buffered saline (PBS) at pH 7.4. With a razor blade, a small cut was made in the cauda epididymis to allow sperm diffusion in the PBS buffer for 5 min. Then, sperm concentrations were determined using 100 μl of the sperm suspension diluted and homogenized in 1.95 ml of PBS. Finally, a 10-μl sample was transferred to the hemocytometer (Improved Neubauer, Deep 0,100 mm, Hausser Scientific, USA) to estimate the total number of spermatozoa in millions/ml (30). Sperm vitality was assessed using eosin-nigrosin stained smears. To the 50 μl of suspension, 1 drop of 3% eosin-Y was added and, after 3 min, 2 drops of 8% nigrosine were added. The percentages of living cells (not stained) and dead cells (stained) were obtained from the 200 spermatozoa analyzed (30). The sperm morphology was evaluated using smears prepared with 10 μl of the sperm suspension, stained with Shorr stain and hematoxylin, and observed in random fields, under a light microscope at x1000. The percentage of 200 spermatozoa morphologically evaluated and classified as normal or abnormal according to Filler (37) was determined.

### Immunohistochemistry for PCNA

Fragments of testes were fixed in 4% paraformaldehyde phosphate-buffered saline (PBS) at pH 7.2 for 24 h. The samples were then washed for 24 h, dehydrated in alcohol, and embedded in a Paraplast Plus^®^ Tissue Embedding Medium (McCormick Scientific, IL, USA). After drying in an oven at 60 °C for 60 min, the 5-μm-thick sections of the testes undergone deparaffinization in xylene and rehydration in graded ethanol solutions. Next, the sections were heated in citrate buffer to 0.01 M at pH 6.0 for 15 min and were blocked with 3% hydrogen peroxide in methanol for 30 min. To block the non-specific reactions, the slides were incubated in a 3% Molico^®^ milk solution (Nestlé, São Paulo, Brazil) for 1 h. The sections were then immunostained with primary polyclonal rat anti-PCNA antibody [#IM-0301] (Imuny, RheaBiotech, Campinas, Brazil) at a concentration of 1 μl/300 μl in PBS buffer and incubated overnight at 4°C. After the primary antibody incubation, the sections were washed with PBS and then incubated with Polymer N-Histofine RAT Multi (Nichirei Biosciences Inc.) for 30 min at room temperature. Finally, the sections were washed with PBS and incubated for 3 min in 50 μl of diaminobenzidine (DAB) solution containing 2 μl of H_2_O_2_. Counterstaining will be performed using hematoxylin. To quantify the PCNA-positive cells, 10 histological fields per animal were captured using the Olympus DP-25 digital camera attached to the Olympus BX41 microscope (Olympus, Tokyo, Japan). The number of points labeled was counted using Olympus CellSens by Dimension with the manual threshold adjustment tool.

### Statistical Analysis

The data normality was verified by the Shapiro-Wilk test and the homogeneity of variances was tested using the Bartlett test. For comparisons of means among the groups, a one-way ANOVA was performed. For the data that violated the homogeneity of the variances, the Brown-Forsythe correction for ANOVA was considered. The Tukey's *post hoc* test was applied if *p* < 0.05. The data were expressed as mean ± SEM. The analyses were performed using the GraphPad Prism^®^ 6.0 software (GraphPad Software, CA, USA).

## Results

### Losartan Does Not Prevent the Reduction of the Testicular and Epididymal Masses 60 Days Post-irradiation

The body weight results showed that there was no significant difference between the experimental groups at 2 and 60 days after the exposure (Table 1). Regarding the reproductive organs, there were no changes in the testes and epididymis wet weights 2 days following exposure to ionizing radiation. On the other hand, a significant reduction of the testes and epididymis wet weights was observed in IR 60 group relative to CTRL 60, which was not avoided by losartan treatment (Table 1). When the male accessory glands were considered, no significant difference was observed after 2 days of the testicular irradiation. The ionizing radiation caused a significant decrease in the seminal vesicle wet weight in IR 60 group after 60 days' exposure though. This reduction was prevented in the animals that received treatment with losartan. No significant alteration was observed in the prostate wet weight among the experimental groups after 60 days post-irradiation (Table 1).

**Table 1:** Average values of the animal's weight, reproductive organs wet weight, and FSH, LH and testosterone levels measured in experimental groups after 2- and 60-days of the exposure to irradiation.

	**2 Days**			**60 days**		
**Parameters**	**CTRL 2**	**IR 2**	**IRLOS 2**	**CTRL 60**	**IR 60**	**IRLOS 60**
Body weight (g)	471.3 ± 16.1	437.8 ± 21.4	436.9 ± 19.0	512.5 ± 21.1	514.3 ± 24.1	476.9 ± 25.4
Testis**·**	3.64 ± 0.19	3.67 ± 0.23	3.95 ± 0.10	3.71 ± 0.10	2.07 ± 0.17^*^	2.24 ± 0.16^*^
Epididymis**·**	1.42 ± 0.04	1.48 ± 0.06	1.54 ± 0.05	1.50 ± 0.03	1.05 ± 0.06^*^	1.15 ± 0.10^*^
Prostate**·**	1.36 ± 0.08	1.23 ± 0.07	1.41 ± 0.13	1.21 ± 0.10	1.04 ± 0.12	1.00 ± 0.08
Seminal vesicle**·**	3.07 ± 0.16	2.87 ± 0.17	3.40 ± 0.19	3.39 ± 0.21	1.96 ± 0.22*#	2.88 ± 0.18
FSH (mlU/mL)	0.08 ± 0.002	0.08 ± 0.002	0.09 ± 0.004	0.10 ± 0.015	0.09 ± 0.005	0.08 ± 0.003
LH (mlU/mL)	0.09 ± 0.002	0.09 ± 0.003	0.10 ± 0.004	0.11 ± 0.008	0.10 ± 0.009	0.10 ± 0.006
Testosterone (ng/mL)	1.57 ± 0.15	1.71 ± 0.18	1.57 ± 0.11	1.25 ± 0.12	1.50 ± 0.20	2.34 ± 0.34^*^

***·**Reproductive organs wet weights are expressed as (g) / body weight of the animal in (kg). Data are expressed as mean ± SEM and analyzed by One-way ANOVA followed by the Tukey test (n = 7/8 for each condition)*.

* *p ≤ 0.05 significant difference relative to CTRL 60*;

*# p ≤ 0.05 significant difference relative to IRLOS 60*.

### Losartan Increases Plasma Testosterone Levels After 60 Days of the Exposure to 2.5 Gy

Our results concerning hormonal determination showed no significant difference in plasma testosterone levels among the groups analyzed 2 days after radiation exposure (Table 1). After 60 days, no difference was observed either between animals submitted to radiation and those in CTRL 60 group. The testosterone levels in IRLOS 60 group were significantly increased though. No significant difference was detected in the serum FSH and LH levels among the studied experimental groups at both study times (Table 1).

### Effects of Ionizing Radiation on Testicular Morphology Are Time Dependent and Losartan Attenuates and Promotes Potential Recovery of Induced Late Damage

**Figure 1:**
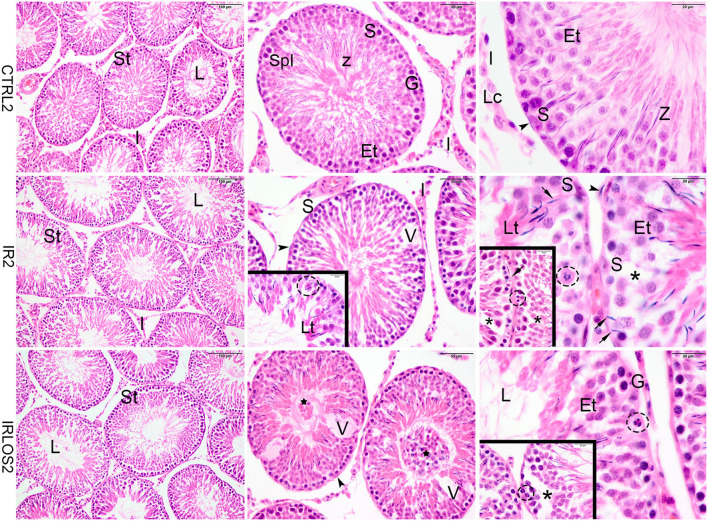
Representative photomicrographs of the testis from control (CTRL 2), irradiated (IR 2), and irradiated and treated with losartan (IRLOS 2) groups 2 days after the exposure, stained with hematoxylin and eosin. In the control group, the testis exhibited a classic architecture and normal seminiferous tubules (St) lined by Sertoli cells (S), which supported germ cells in different stages of differentiation. Pattern interstitial tissue (I) with Leydig cells (Lc) aggregates was also observed. In the irradiated groups, independently of the treatment, seminiferous tubules exhibited damage signal irradiation, characterized by loss of germ cells (*), presence of vacuoles (V), germ cells with pycnotic nucleus (dashed circle), suggesting apoptotic death and sloughing of cells into the lumen (⋆). Note retention and phagocytose of step-19 spermatids in Sertoli basal cytoplasm (→). Et, early spermatids; G, spermatogonia; Lt, late spermatids; L, lumen; SpI, primary spermatocytes; Z, sperm. ▸ = peritubular myoid cells. Scale bar, first column (100μm), second column (50 μm), and third column and insets (20 μm).

**Figure 2:**
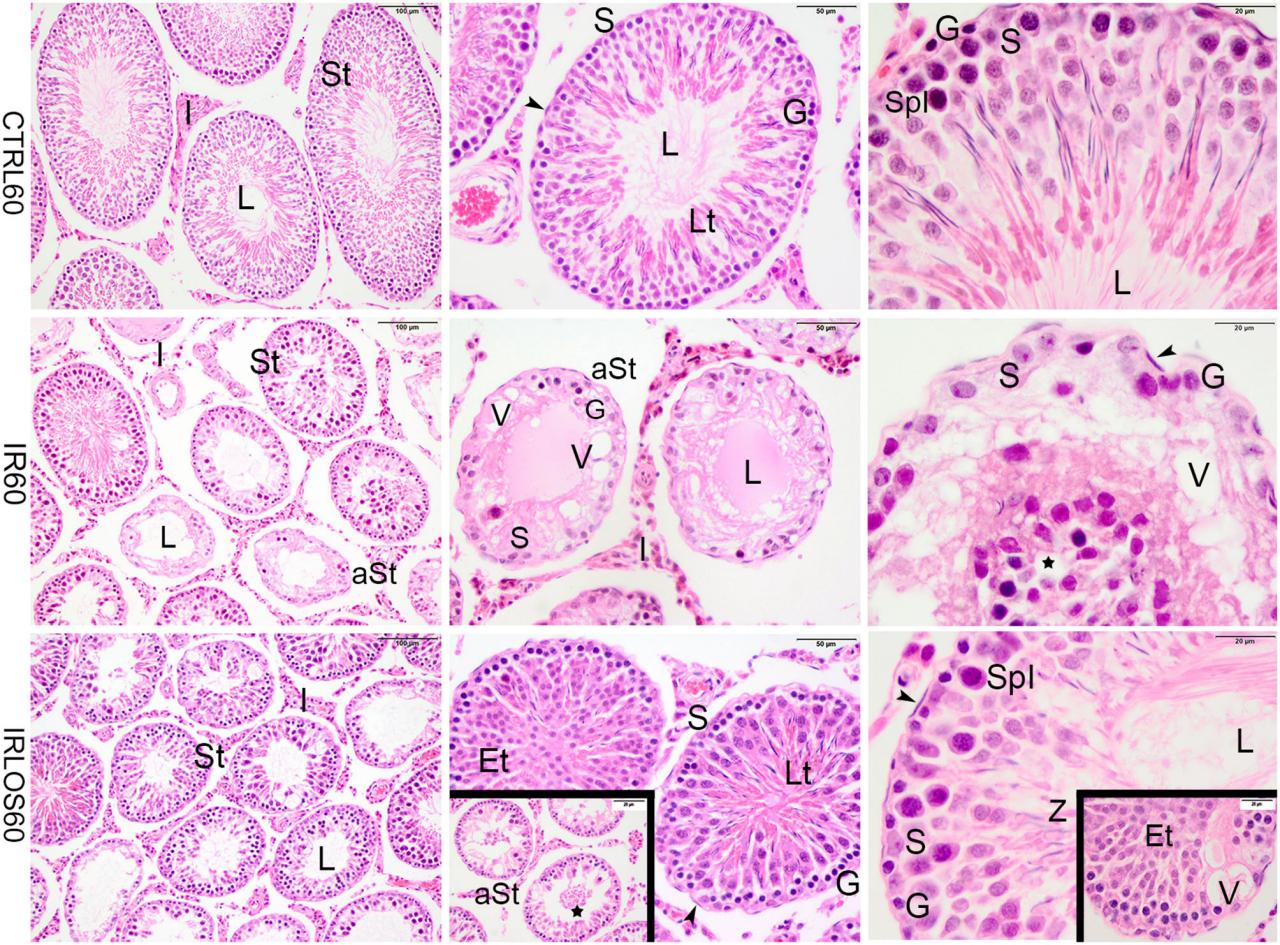
Representative photomicrographs of the testis from control (CTRL 60), irradiated (IR 60), and irradiated and treated with losartan (IRLOS 60) groups 60 days after the exposure, stained with hematoxylin and eosin. In the control group, the testis exhibited a classic morphology and normal seminiferous tubules (St), lined by Sertoli cells (S), with germ cells in different stages of development. In the irradiated group, seminiferous tubules exhibited damage signal irradiation, characterized by loss of germ cells (*), presence of vacuoles (V), atrophied seminiferous tubules (aSt), and sloughing of cells into the lumen (⋆). Note the Sertoli cells and spermatogonia were the unique surviving cells in the seminiferous epithelium. In the irradiated and treated group, seminiferous tubules in recovering were observed with scattered degenerated tubules. The recovery tubules exhibit seminiferous epithelium with germ cells in advanced stages of spermatogenesis. Et, early spermatids; G, spermatogonia; Lt, late spermatids; L, lumen; SpI, primary spermatocytes; Z, sperm; ▸, peritubular myoid cells; Scale bar, first column (100μm); second column (50 μm); and third column and insets (20 μm).

The histopathological analysis of the testes harvested from the control group in both post-irradiation times (2 and 60 days) revealed a classic histological architecture, with seminiferous tubules showing the cyclical process of spermatogenesis. The seminiferous tubules presented central lumen and seminiferous epithelium, which contained concentric layers of germ cells. In the tubular periphery, the nucleus of the Sertoli cells exhibited a normal shape with an evident nucleolus. Peritubular or myoid cells were found to be surrounding the seminiferous epithelium and were separated from this epithelium by a basal membrane. Leydig cell aggregates with a normal morphologic pattern were observed in interstitial tissue, near blood vessels (Figures 1, 2, first lines).

In the irradiated groups, the degree of damage to the seminiferous tubules varied according to the time post-irradiation. Then, in the animals exposed to 2.5 Gy and analyzed at 2 days of the exposure, the seminiferous tubule morphology showed a regular organization similar to the control group, with differentiating germ cells. Signals of damage have already been observed in the seminiferous epithelium though, characterized by vacuoles due to the loss of germ cells and disaggregation of germ cells, which indicate breakage of the cytoplasmic bridges, resulting in the sloughing of germ cells into the lumen. Retention and phagocytosis of step-19 spermatids in Sertoli basal cytoplasm, and germ cells with a pycnotic nucleus, suggesting apoptotic death were also observed (Figure 1, second line). The treatment with losartan for 2 days after the irradiation was not sufficient to mitigate the acute damage observed (Figure 1, third line).

In contrast, the testes of rats analyzed at 60 days post-irradiation showed significant degenerative effects of ionizing radiation marked by the total disruption of the germinal tissue. The seminiferous tubules were completely disrupted, atrophied, and presented significant structural impairment. The seminiferous epithelium exhibited severe loss of germ cells and the space was occupied by numerous vacuoles of varying shapes. In depleted tubules, the Sertoli cells and a few spermatogonia were the unique surviving cells in the seminiferous epithelium. No evidence of spermatogenic activity or spermatozoa was observed in these tubules. Nevertheless, seminiferous tubules exhibiting lesser signals damage were observed interspersing the degenerated tubules. In this case, the germinative epithelium tubular evidenced cells in different phases of spermatogenesis. Evident interstitial tissue expansion relative to the seminiferous tubules was observed, possibly due to the intense tubular atrophy (Figure 2, second line).

In the group that received the treatment with losartan by 60 days post-irradiation, several seminiferous tubules were observed in recovery. The germinative epithelium of the recovery tubules exhibited cellular reorganization, and germ cells differentiated in advanced stages of spermatogenesis, although slight signs of damage such as vacuoles were still present. In addition, the recovering tubules were observed with scattered degenerated tubules (Figure 2, third line).

### Treatment With Losartan Improves Late Damage in Epididymis Post-irradiation of the Testes

**Figure 3:**
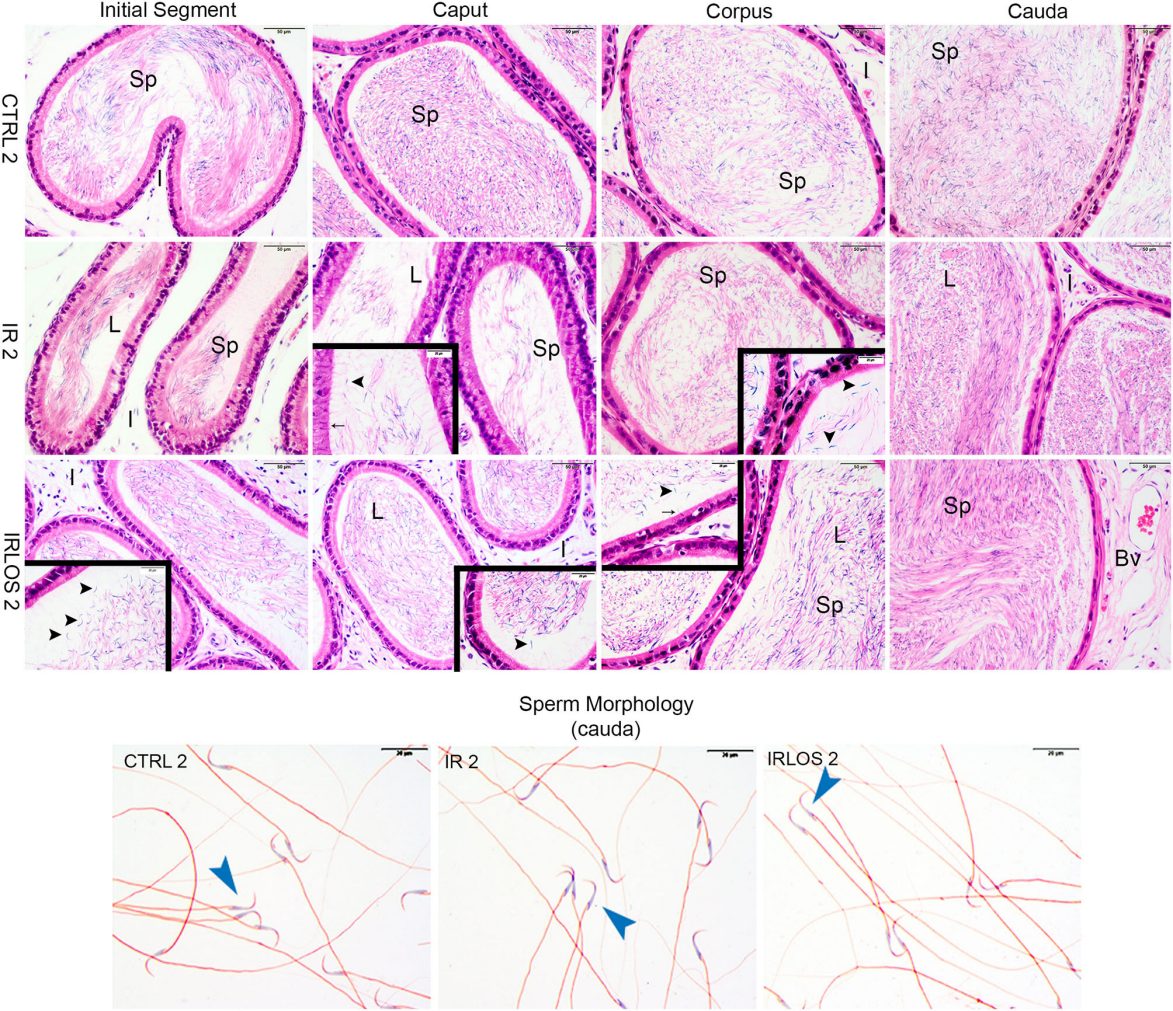
Representative photomicrographs of epididymis and sperm morphology from control (CTRL 2), irradiated (IR 2), and irradiated and treated with losartan (IRLOS 2) groups after 2 days of exposure to the ionizing radiation. In the control group, the epididymis segments exhibited a classic morphology and the lumen showed high sperm (sp) content. Pattern interstitial tissue (I) without inflammatory infiltrate was observed. In the irradiated groups, independently of the treatment with losartan, the epididymis segments (initial segment, caput, corpus, and cauda) exhibited histological characteristics similar to the control, with high intraluminal sperm density (L). No evidence of inflammatory infiltrate was observed in the interstitial tissue. An apparent damage signal in some tubules was the presence of isolated heads of sperm (▸) and phagocytosis of sperm (→). Isolated sperms from cauda of epididymis exhibited normal forms in all analyzed groups. Bv, blood vessels; ▸, sperm with normal morphology. Scale bar, 50 μm, insets, 20 μm. Staining: Epididymis, hematoxylin and eosin; Sperm, Shorr.

**Figure 4:**
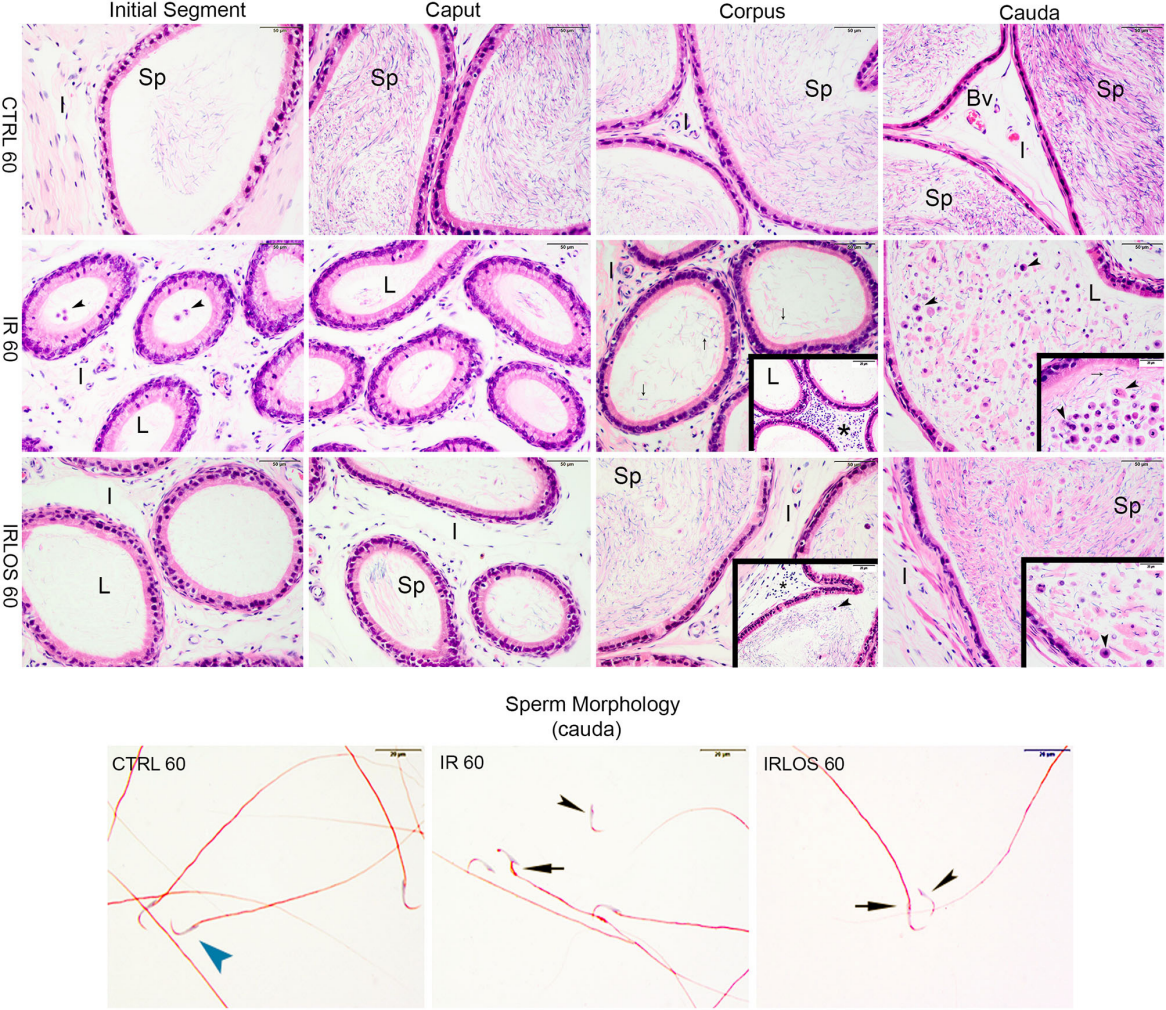
Representative photomicrographs of epididymis and sperm morphology from control (CTRL 60), irradiated (IR 60), and irradiated and treated with losartan (IRLOS 60) groups after 60 days of exposure to the ionizing radiation. In the control group, the epididymis segments (initial segment, caput, corpus, and cauda) exhibited a normal morphology, with high intraluminal (L) sperm (sp) content. The irradiated group exhibits damage signal irradiation, characterized by a decrease of sperm content and extensive exfoliated germ cells and cells debris (▸) from the testes. Interstitial tissue (I) exhibited inflammatory infiltrates (*), being most frequently seen in the corpus. In the irradiated and treated group, losartan attenuated the damage to the epididymis. The interstitial tissue presented a slight inflammatory infiltrate. Reduced density of exfoliated germ cells and cell debris was observed, and normal forms of sperm into the lumen, although isolated sperm head and tail were still frequent. In irradiated groups, isolated sperm from cauda of epididymis exhibited abnormal morphology. Bv, blood vessels. ▸ sperm with normal morphology; ▸, head defects; →, midpiece defects; scale bar, 50 μm, insets, 20 μm. Staining: Epididymis, hematoxylin and eosin; Sperm, Shorr.

The analysis of the epididymis segments (initial segment, caput, corpus, and cauda) from the control groups after 2 and 60 days of the exposure showed a pattern of histological architecture. The lumen exhibited high sperm content. Similarly, there was no any morphologic change in the interstitial tissue (Figures 3, 4, first lines).

After 2 days of exposure, the epididymis from IR 2 and IRLOS 2 showed histological characteristics similar to the control, with high intraluminal sperm density. In addition, there was no evidence of inflammatory infiltrate. Nevertheless, some tubules exhibited isolated heads of sperm and phagocytosis of sperm (Figure 3, second and third lines)

On the other hand, the histopathological analysis evidenced that the ionizing radiation induced leukocyte infiltration in interstitial tissue of the epididymis, mainly in the corpus, 60 days after the exposure. The intraluminal content was gradually increasing from the initial segment to the cauda, which was predominantly characterized by isolated sperm heads and tails, exfoliated germ cells, and cell debris from the testis (Figure 4, second line).

The treatment with losartan attenuated the damage to the epididymis that were observed 60 days post-irradiation. In these animals, the interstitial tissue of epididymis, from its caput segment, but mainly in the corpus, presented a slight inflammatory infiltrate. Regarding the intraluminal content, reduced densities of exfoliated germ cells and cell debris were observed, in addition to normal forms of sperm. Nevertheless, isolated sperm head and tail were still frequent (Figure 4, third line).

### After 60 Days of Irradiation, Treatment With Losartan Can Attenuate the Increase of the Vacuolar Density

The morphometric data of the testes showed that there was no difference in seminiferous tubules diameter between the groups studied, 2 days after irradiation. The tubular diameter was reduced 60 days after irradiation and losartan treatment was not able to revert that though (Table 2). The stereological analysis evidenced a significant reduction in seminiferous tubule density, in parallel with an increase in the interstitial tissue, observed in the testicular parenchyma already 2 days after exposure to radiation. These changes that persisted for 60 days after irradiation were not reverted by treatment with losartan. An increase of vacuole density in the seminiferous epithelium was also observed already 2 days of irradiation, which was even higher following treatment with losartan, reaching statistical significance regarding CTRL 2 group. Similarly, the vacuolar density was drastically increased by radiation 60 days after. Notably, in the presence of losartan, this vacuolar density elevation was lower, although not significantly (Table 2).

**Table 2:** Tubular diameter, density of the seminiferous tubules, interstitial tissue, and vacuoles in testicular parenchyma, and sperm evaluation of the animals of each experimental group after 2- and 60-days of the exposure.

	**2 days**			**60 days**		
**Parameters**	**CTRL 2**	**IR 2**	**IRLOS 2**	**CTRL 60**	**IR 60**	**IRLOS 60**
Tubular diameter (μm)	265.9 ± 7.96	246.2 ± 5.61	241.3 ± 7.15	273.1 ± 5.10	189.8 ± 8.50^*^	204.8 ± 8.80^*^
Tubular density (%)	77.52 ± 4.01	66.01 ± 2.51^*^	64.36 ± 3.24^*^	65.40 ± 1.80	52.80 ± 2.04^*^	51.43 ± 1.95^*^
Interstitial density (%)	22.14 ± 4.00	33.10 ± 2.53^*^	34.20 ± 3.23^*^	34.58 ± 1.80	44.39 ± 1.71^*^	46.60 ± 1.82^*^
Vacuolar density (%)	0.34 ± 0.18	0.89 ± 0.29	1.44 ± 0.29^*^	0.02 ± 0.01	2.81 ± 0.60^*^	1.97 ± 0.43^*^
Sperm concentration (x10^6^/ml)	57.73 ± 7.07	54.62 ± 8.53	60.46 ± 9.98	48.96 ± 6.19	7.262 ± 1.51^*^	11.16 ± 3.44^*^
Sperm vitality (%)	100 ± 0.0	99.75 ± 0.25	99.38 ± 0.42	98.69 ± 0.57	57.13 ± 11.80^*^	76.31 ± 9.89
Sperm morphology (%)	97.19 ± 0.60	94.81 ± 1.05	96.50 ± 0.78	97.13 ± 0.29	77.43 ± 5.10^*^	77.63 ± 5.35^*^

*Data are expressed as mean ± SEM and analyzed by One-way ANOVA followed by the Tukey test (n = 7/8 for each condition)*.

* *p ≤ 0.05 significant difference relative to CTRL group within the same time*.

### Sperm Vitality Is Partially Protected by Treatment With Losartan After 60 Days of 2.5 Gy Irradiation

The results of sperm evaluation showed that the impact of exposition to 2.5 Gy of ionizing radiation on sperm occurred only after 60 days (Table 2). In these animals, sperm concentration was significantly reduced in IR 60 and IRLOS 60 groups. A parallel reduction in the percentage of sperm with normal morphology following radiation exposition, independently of the treatment with losartan, was also observed. On the other hand, the significant reduction in the percentage of live sperm observed in the animals of the IR 60 group was prevented by treatment with losartan (Table 2). The radiation caused sperm defects mainly after 60 days of the exposure, which were characterized by separation of head and tail (loose head), small head, head with a smaller acrosomal curvature, and midpiece defect as asymmetrical insertion of the midpiece (Figures 3, 4, fourth lines).

### Treatment With Losartan Accelerates the Cellular Proliferation in Irradiated Testes After 60 Days

**Figure 5:**
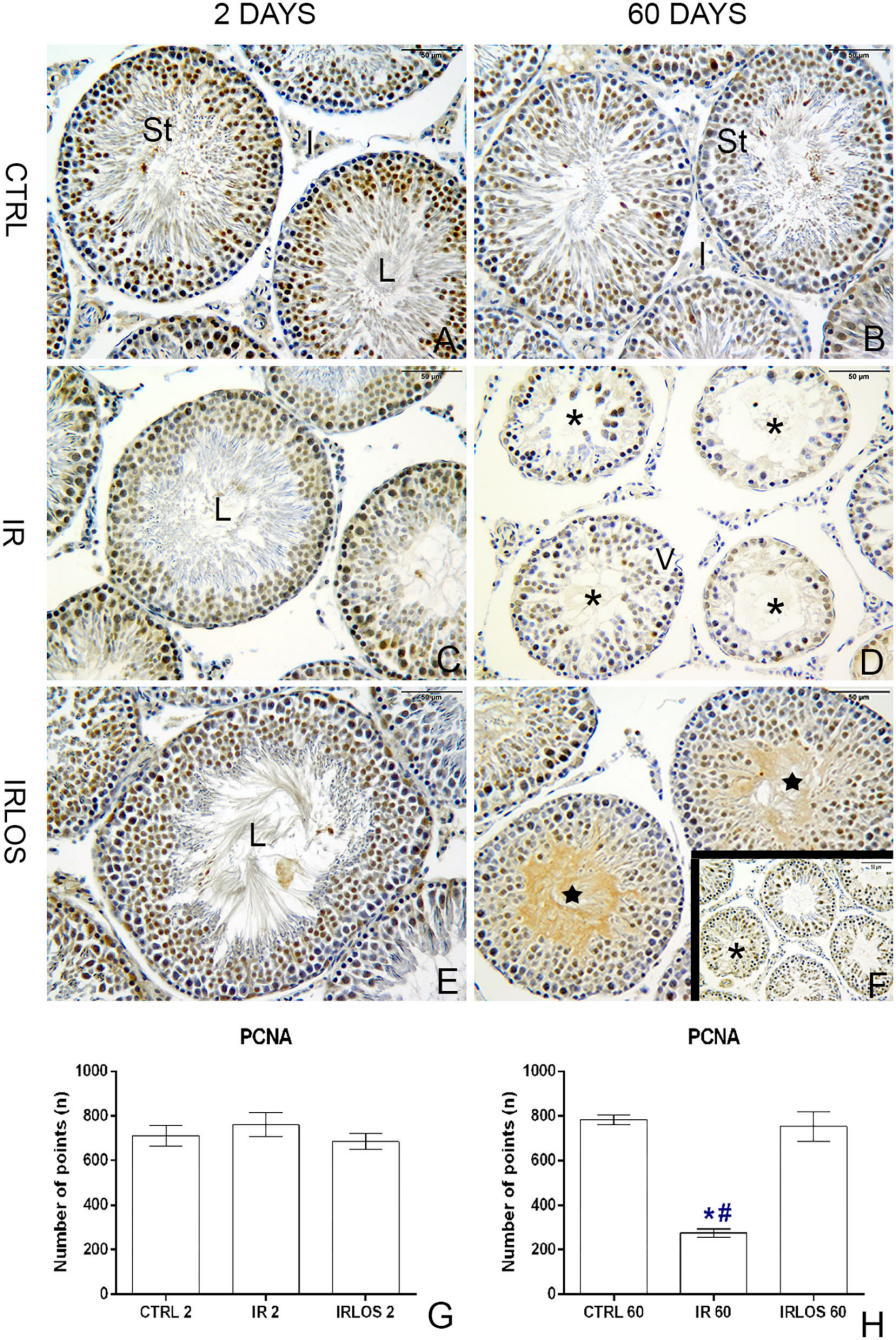
PCNA immunohistochemistry of the testes from controls **(A,B)**, rats exposed only to irradiation **(C,D)**, and rats exposed to radiation and treated with losartan **(E,F,F-inset)** after 2 and 60 days to the exposure. Positive reactions were observed in many nuclei of seminiferous tubule cells of the testes from controls. Depending on the spermatogenesis stage, different types of spermatogonia and spermatocytes presented PCNA-positive labels. In the irradiated group, PCNA-positive cells were observed in only nuclei of surviving spermatogonia. In rats that were both irradiated and treated, positive reactions were observed in many nuclei of spermatogonia and spermatocytes of seminiferous tubules with signs of recovery. PCNA-positive cell quantification **(G,H)**, 2 and 60 day post-irradiation, respectively. Immunoperoxidase staining is in brown. Counterstained with hematoxylin, which stains the nucleus is in blue. Scale bar, 50 μm. Data expressed as means ± SEM. One-way ANOVA was followed by the Tukey test. ^*^p ≤ 0.05 significant difference in relation to CTRL 60 group; # *p* ≤ 0.05, significant difference in relation to IRLOS 60 group. Sample size = 6.

In rats' testes assessed 2 days post-irradiation, there were no differences in PCNA immunostaining between groups (Figures 5A,C,E,F). On the other hand, there was a significant PCNA immunostaining reduction in the rat testes 60 days following irradiation. Notably, these PCNA immunostainings when present were observed mainly in surviving spermatogonium. The treatment with losartan for 60 days significantly increased the PCNA-positive cells in the seminiferous epithelium though. These labels were detected in many spermatogonia and spermatocytes of tubules preseting signs of recovery of the seminiferous epithelium (Figures 5B,D,F,H).

## Discussion

The ionizing radiation changed neither the body mass nor the moist masses of reproductive organs 2 days after exposure, which is in accordance with other studies that also employed acute irradiation protocols (38–40). Despite this, the histopathological evaluation of the testes revealed damage to the germinal tissue characterized by the apoptosis of germ cells, with consequent formation of vacuoles in the seminiferous epithelium, sloughing of germ cells into the lumen, as well as retention and phagocytose of step-19 spermatids in Sertoli basal cytoplasm. In addition, this damage was also manifested by a reduction in the seminiferous tubule's density, which occurred with an interstitial tissue increase, without impacting the tubular diameter.

This acute radiation-induced damage to the reproductive tissue indicates a direct cytotoxic action on the testis, which caused apoptosis of specific germ cell types, as well as specific disturbances on Sertoli cell function. This can be inferred by the finding of germ cell exfoliation, which may have as potential causes the disruption of Sertoli and germ cell junctions and of Sertoli cytoskeletal fibers.

The presented data also showed retention and phagocytosis of step-19 spermatids in Sertoli basal cytoplasm, which indicates disturbance of Sertoli cell function. This finding may also be related to the impairment of both testosterone secretion and spermatid development (34, 41). Mature spermatozoa release into the seminiferous tubule lumen is an androgen-dependent process, which involves intense modification of adhesion molecules at the interface between Sertoli cells and spermatozoa, resulting in loss of binding between these cells and, consequently, spermiation (42). In case of androgen signaling changes, mature sperm that should be released during stage VIII from the seminiferous epithelium would be phagocytosed by Sertoli cells and then kept in their basal cytoplasm (41, 43). In this study, there was no change in testosterone levels or in FSH and LH hormones between the groups studied 2 days after irradiation though. This suggests that testosterone secretion is affected neither by direct radiation effects on Leydig cells nor endocrine-mediated consequences within this period of time. Thus, the retention and phagocytosis of step-19 spermatids that were observed in animals exposed to irradiation may reflect a possible dysfunction in the Sertoli cells or in the differentiation of round spermatids, which needs further confirmation.

Despite the histopathological analysis showing damage to the seminiferous epithelium, the data on sperm concentration, vitality, and morphology did not reveal significant changes 2 days after irradiation. This is an expected finding considering the epididymal transit time since the sperm present in the epididymis depict events that took place in the testes about 2 weeks in advance (35, 44). Thus, it is expected that the deleterious effects of radiation on gametes are first observed in the seminiferous tubules and, later, in the epididymal caput, but not yet in the cauda. Actually, spermatozoa that are in the ducts of the epididymis caput reflect events that occurred previously in the testes, about 2 to 5 days. On the other hand, spermatozoa that are in the epididymis cauda show normal patterns of concentration, vitality, and morphology, as they are cells from a spermatogenic cycle prior to the action of radiation (34, 41). The analysis of the epididymal segments (caput, corpus, and cauda) made after 2 days of exposure has confirmed this hypothesis.

Notably, all the above-mentioned short-term effects of radiation on the testes that occurred 2 days after exposure were not prevented or mitigated by treatment with losartan. It indicates that this treatment was not able to blunt the direct action of radiation on the gonadal tissue. Nevertheless, the increased density of vacuoles in the germinal epithelium observed in animals treated with losartan, 2 days after irradiation, suggests an effect of this RAS inhibitor on the activity of Sertoli cells. Low to moderate doses of radiation lead cells to apoptotic death and tissues with high proliferative activity, such as the testes, are associated with increased susceptibility to apoptosis (8). In as much as the cell death progresses, the Sertoli cell phagocytizes the apoptotic cell, which quickly makes any evidence of this process disappear (41, 42). It is noteworthy, however, that the proliferation of type A spermatogonia does not occur at the same speed in order to repair this damage. Thus, considering that germ cell apoptosis starts minutes after irradiation and the number of apoptotic cells increases until approximately 16 h after irradiation (39, 45), it can be suggested that treatment with losartan may accelerate the removal of dead cells and residual bodies by Sertoli cells, resulting in the formation of a greater number of vacuoles in the seminiferous epithelium. This hypothesis, however, requires further confirmation.

Sixty days after exposure, the deleterious effects of ionizing radiation were evident in spermatogenesis and were characterized by reduction of testicular, epididymal, and seminal vesicle masses; induction of tubular atrophy; increment in the density of interstitial tissue and vacuoles in the testicular parenchyma; and reduction of cellular proliferation. Further, reduced spermatozoa production and augmented abnormal spermatozoa in addition to reduced sperm content associated with the exfoliated germ cells in the epididymal lumens confirmed germ cell degeneration. This evidence suggests a progressive loss of germ cells over the course of 60 days, as a result of increased radiation-induced oxidative stress. Similar, but even more intense damage was previously obtained in the rat testes submitted to a higher radiation dose (5.0 Gy) (30).

The present data show that losartan significantly improved certain parameters such as preventing the reduction of seminal vesicle mass, increasing sperm vitality, reducing vacuoles number, and increasing cell proliferation in the seminiferous epithelium 60 days after irradiation. This treatment also increased the sperm content and reduced the exfoliated germ cells in epididymal lumens in addition to increased circulating testosterone levels. The histopathological analysis of the testes as well as of the corresponding epididymis reinforces a potential gonadal restructuring and recovery of spermatogenesis in the animals treated with losartan. In fact, several tubules showed signs of recovery in their germinal epithelium. These signals suggest an initial reorganization by Sertoli cells and spermatogonia, as well as the resumption of the differentiation capacity of the seminiferous epithelium, due to the presence of cells in advanced stages of spermatogenesis such as spermatocytes, spermatids, and even sperm. Moreover, there was immunostaining for PCNA in several spermatogonia and dividing spermatocytes in the seminiferous tubules of irradiated animals that were treated with losartan, reinforcing the intensification of cell proliferation. Therefore, the regeneration of seminiferous tubules was characterized by a progressive increase in proliferative capacity and differentiation of germ cells, suggesting that losartan may have accelerated the resumption of spermatogenesis.

An important axis whereby RAS regulates the testicular functions is the Ang II/AT1R axis. Ang II is the main effector peptide of the RAS in the male reproductive system, and it is present in both germ cells and Leydig cells (24, 26). Through this axis, Ang II may promote negative regulation of testosterone production by inhibition of the adenylate cyclase activity in rat Leydig cell membranes due to the reduced gonadotropin-stimulated cAMP (26, 46). In addition, AT1R is present in germ cells at different maturation stages, and its involvement in the regulation of spermatogenesis has been suggested (47, 48). Considering that Ang II produced in the testes modulates steroidogenesis in Leydig cells through AT1R, blocking this pathway is likely related to the potential radioprotection mechanisms of losartan. The inhibition of this pathway may upregulate testosterone production, thereby favoring the recovery of spermatogenesis. The increased plasma testosterone levels as well as the preservation of the moist mass of the seminal vesicle, a testosterone-dependent organ, in irradiated animals that were treated with losartan reinforce the hypothesis. Curiously, neither irradiation nor treatment with losartan influenced the mass of the prostate, another androgen-dependent gland.

In irradiated tissues, it is also important to highlight the interaction between ionizing radiation, oxidative stress, and RAS, which may be involved in the progression of the indirect effects of radiation and changes in cell function and phenotype, leading to a chronic inflammatory state (16, 23). The involvement of Ang II and NADPH oxidase in the elevation of ROS production after radiation exposure may then lead to the chronicity of oxidative stress and RAS activation in the irradiated tissue (14, 15, 17). Thus, the use of losartan and consequently blocking of the Ang II/AT1R axis may induce upregulation of testosterone production and, possibly, contribute to a decrease in the generation of ROS *via* activation of NADH oxidase.

Interestingly, the inhibition by losartan of the Ang II/AT1R axis 60 days after irradiation did not exert any influence on the serum LH and FSH levels. In a previous study that investigated the role of AT1R on spermatogenesis and expression of hormones from the hypothalamic-pituitary-gonadal (HPG) axis in both wild C57BL/6 and ± AT1 knockout mice, it was demonstrated that losartan had no significant effect on the LH and FSH levels (48). However, 180 days after exposure to a single dose of radiation (9 Gy) on the testes, no change in FSH levels was observed until the 23rd day post-irradiation (49). In this study, a significant elevation of FSH levels was observed between the 34th and 118th days post-irradiation. On the other hand, LH levels increased after the 34th day post-irradiation, which was significant until the 71st day, remaining so until the end of the experiment. In this period, there were no significant changes in plasma testosterone levels (49). These authors proposed the influence of the developmental stages of germ cells on Sertoli cell function and assume that androgen-binding protein (ABP) levels decreased on the 15th day, when the degeneration of the seminiferous epithelium reached the pachytene spermatocytes, while FSH and LH levels only increased when degeneration reached the elongating spermatids.

Considering that in this study, the radiation dose was almost four times lower than that used by Pinon-Lataillade and co-authors (49), in the testes of the irradiated animals, although having completely depleted tubules, it was possible to observe surviving germ cells at different stages of spermatogenesis. This suggests that the permanence of these cells in the seminiferous tubules may have contributed to the lack of alteration of FSH and LH levels. Additionally, in irradiated animals that received losartan, the larger-scale tubular recovery also did not change the pituitary axis. In addition, Zhao and coauthors' (2021) data have already shown that only losartan does not influence this HPG axis.

Furthermore, this study showed the radioprotective effects of losartan when the employed radiation dose, administered directly to the testes, was close to the ED50. This result is related to the kinetics of spermatogenesis decline and recovery (5, 50). After exposure to a cytotoxic agent, such as ionizing radiation, patterns of recovery can be observed depending on the degree of death of the stem cells: (1) if no stem cells die, the sperm production returns to normal; (2) if any stem cell dies, the recovery of sperm production may or may not be complete; (3) if all stem cells die, azoospermia may be permanent. Thus, the data from this study suggest that by administering an intermediate dose of irradiation directly into the scrotum of the animals, associated with the treatment with losartan, the chances of survival of stem cells were potentiated and, with that, their regeneration and, subsequently, repopulation of seminiferous tubule from the resumption of spermatogenesis. Our previous study using the same experimental model, but exposing the testes to a dose of 5.0 Gy, reinforces that the kinetics of spermatogenesis is fundamental in the search for substances with radioprotective effects as, at this dose, testicular recovery was extremely compromised by excessive death of stem cells (30).

Although some parameters reinforce the radioprotective effects of losartan in rat tests, others failed to show a progressive evolution in the face of damage caused directly by irradiation and as a result of oxidative stress. Among the recovering seminiferous tubules in the testicular parenchyma, tubules with degenerative signs and vacuoles were still present. Parameters such as reduced testicular and epididymal mass, tubular atrophy, and both reduced sperm concentration and sperm with normal morphology did not undergo damage reversal. Perhaps, a longer losartan treatment in these animals could show a clearer testicular recovery. This hypothesis is reinforced by Abuelhija et al., (2013) that suggest that the Wistar lineage possibly requires a longer time for recovery of the germinal epithelium after radiation, thus surpassing a spermatogenic cycle, which in rats is 58–60 days.

In conclusion, our data indicate that treatment with losartan prevents certain effects that occur 60 days after radiation exposure without, however, having much influence upon those that occur 2 days after exposure. Among these radioprotective actions of losartan, emphasis should be placed on the prevention of reduction of seminal vesicle mass, improvement in sperm vitality, elevation of plasma testosterone levels, reduction of vacuoles number, and increment of cell proliferation in the seminiferous epithelium, in addition to seminiferous tubules with a strong recovery of the seminiferous epithelium. This suggests that inhibition of the AngII/AT1R axis by losartan is more effective in protecting the indirect/delayed radiation damage, resulting from the oxidative stress introduced in the tissue.

Considering that the action radioprotector of the losartan on the certain damage to the testes is no guarantee that the fertile capacity will be preserved, it remains to be seen whether this drug can also act in the protection of the sperm genetic integrity which is essential for preserving fertility.

Thus, a new perspective emerges for radiotherapy treatment, using higher doses of radiation, considering the improvement of radioprotection in healthy testicular cells. This proposal is also supported by other preclinical studies that show the efficacy of RAS inhibitors to prevent radiation-induced damage to healthy extra-testicular tissues without, however, impairing the tumor response to radiation [55]. In addition, considering that several angiotensin-converting enzyme inhibitors or AT1R antagonists are routinely used in the treatment of cardiovascular dysfunctions, all of which are well tolerated and safe, a promising perspective of radioprotection in the testis opens, as the results of the present project could be especially interesting for male patients who are submitted to radiotherapy for pelvic cancer in reproductive age, as it may contribute to a better quality of life after treatment.

## Data Availability Statement

The original contributions presented in the study are included in the article/supplementary material, further inquiries can be directed to the corresponding author/s.

## Ethics Statement

The animal study was reviewed and approved by Ethics Committee for the Use of Experimental Animals from the Marília Medical School (CEUA/FAMEMA, protocol number 1525/19).

## Author Contributions

MS, AC, and MA designed the research study. LS, AC, and MS performed the research. LS and MS analyzed the data. MS wrote the paper. MA and AC performed the analysis with constructive discussions as well as reviewed the writing. All authors contributed to the article and approved the submitted version.

## Funding

Financial support for this study was provided by the Brazilian Agency - São Paulo Research Foundation (FAPESP) through a Regular Research Grant [Grant No. 2019/09488-2, Principal Investigator MS and Collaborators MA, and AC] and by “Fundação para a Ciência e a Tecnologia”—FCT to UMIB (UIDB/00215/2020 and UIDP/00215/2020) and the ITR—Laboratory for Integrative and Translational Research in Population Health (LA/P/0064/2020). This study was partially financed by the Coordination for the Improvement of Higher Education Personnel - Brazil (CAPES) - Finance Code 001 and provided scholarship funding for LS [Supervisor MS].

## Conflict of Interest

The authors declare that the research was conducted in the absence of any commercial or financial relationships that could be construed as a potential conflict of interest.

## Publisher's Note

All claims expressed in this article are solely those of the authors and do not necessarily represent those of their affiliated organizations, or those of the publisher, the editors and the reviewers. Any product that may be evaluated in this article, or claim that may be made by its manufacturer, is not guaranteed or endorsed by the publisher.
